# TFEB-mediated autophagy stimulation as an anabolic strategy for bone: insights from TFEB activation in the osteoblast lineage

**DOI:** 10.1080/27694127.2025.2596422

**Published:** 2025-12-08

**Authors:** Melda Onal

**Affiliations:** a Department of Physiology and Cell Biology, University of Arkansas for Medical Sciences, Little Rock, AR, USA; b Center for Musculoskeletal Disease Research (CMDR), University of Arkansas for Medical Sciences, Little Rock, AR, USA

**Keywords:** Autophagy, bone, bone anabolic, bone formation, osteoblast, osteoprogenitor TFEB

## Abstract

Autophagy in the osteoblast lineage is essential for bone formation and skeletal homeostasis, yet the mechanisms through which it supports bone formation remain unclear. To investigate these mechanisms and evaluate the anabolic potential of autophagy stimulation, we generated a genetic mouse model in which transcription factor EB (*Tfeb*), a master regulator of autophagy and lysosomal biogenesis, was elevated specifically in osteoblast-lineage cells. *Tfeb* elevation increased the expression of autophagy and lysosomal genes and enhanced autophagic flux in osteoblasts. Stimulation of autophagy increased bone formation in both cortical and cancellous bone compartments, leading to gains in bone mass and strength. Single-cell RNA sequencing revealed reduced osteoblast apoptosis, suggesting improved cell survival as a contributor to the observed increase in osteoblast number. Our ex vivo studies also suggest that autophagy stimulation increases proliferation of osteoblats lineage cells. In addition to increasing osteoblast number, *Tfeb* elevation also enhanced osteoblast function, likely by increasing transcription and translation of extracellular bone matrix components. Taken together, these findings demonstrate that elevation of *Tfeb* in the osteoblast lineage cells stimulates autophagy, promotes bone formation, and leads to increased bone mass and strength, supporting further investigation of TFEB or autophagy activation as a potential therapeutic strategy for osteoporosis.

## Punctum

Throughout life, bones are remodeled by cells that resorb bone (osteoclasts) and cells that form bone (osteoblasts). Reduction in the production or function of osteoblasts can shift the balance between bone resorption and formation, leading to bone loss. Autophagy is one of the cellular mechanisms that supports bone formation. Elimination of autophagy from different stages of the osteoblast lineage, i.e., progenitors, osteoblasts, and osteocytes, has revealed that autophagy is essential throughout this lineage as its inhibition at any stage decreases osteoblast number and bone formation. However, the molecular mechanisms through which autophagy supports bone formation, and the therapeutic value of autophagy stimulation for the skeleton remains unclear.

To start addressing these questions, we created a genetic mouse model in which we stimulated autophagy in osteoblast lineage cells by increasing the expression of transcription factor EB (*Tfeb*)[1]. TFEB regulates genes involved in autophagy and lysosomal biogenesis. Accordingly, increasing TFEB increased expression of autophagy and lysosomal genes in bone, which was sufficient to stimulate autophagy in osteoblasts.

Bones are composed of a dense outer compartment (cortical bone), and a lattice-like inner bone compartment (cancellous bone). These two compartments are produced and maintained differently, and they provide different contributions to mechanical and hormonal demands. *Tfeb* elevation increased bone formation in both bone compartments. Histomorphometry revealed that increases in bone formation were due to increased osteoblast number and function. *Tfeb*-induced bone formation led to progressive gains in bone mass, which were associated with increases in cortical thickness and cancellous bone volume. Overall, these changes augmented bone strength.

Autophagy is commonly thought of as a stress response mechanism that ensures cell survival and function. Therefore, we were somewhat surprised by the anabolic impact of autophagy stimulation in young healthy mice. One interpretation of our results is that even under physiological conditions, osteoblast number and bone formation are constrained by cellular stressors. Indeed, when we performed single-cell RNA sequencing (scRNA-seq) and gene ontology analysis, we observed a decrease in apoptosis in osteoblasts of authophagy stimulated mice. Reduced apoptosis alone could be sufficient to increase osteoblast number, bone formation, and bone mass. Thus, *Tfeb*-induced autophagy may enhance osteoblast survival to increase bone formation.

One stressor limiting osteoblast survival and function could be endoplasmic reticulum (ER) stress. Because osteoblasts are highly secretory and their main product, collagen, is prone to misfolding, they are thought to experience a tonic level of ER stress. We therefore hypothesized that elevating *Tfeb*, and thereby increasing autophagy, would reduce this tonic ER stress to improve osteoblast survival and function. Contrary to this hypothesis, scRNA-seq analysis did not reveal a significant reduction in ER stress gene markers in osteoblasts following *Tfeb* elevation. Instead, *Tfeb* elevation increased transcription of extracellular matrix components and translation machinery (including ribosomal RNAs [rRNAs]). Taken together, these findings indicate that under physiological conditions, *Tfeb* elevation does not act by alleviating ER stress. Rather, it appears to promote osteoblast function by enhancing matrix production at both transcriptional and translational levels.

As autophagy does not directly modulate transcription, the observed increases in matrix genes and translational machinery must be indirect. One possible explanation is that osteoblasts exhibit a tonic level of integrated stress response (ISR) rather than ER stress alone and stimulating autophagy dampens this pathway. The ISR is a central signaling network that responds to stress conditions (e.g. protein misfolding, nutrient deprivation, oxidative stress) by reprogramming translation. Similar to observations in a murine model of osteogenesis imperfecta, it is possible that naturally misfolded collagen activates ISR in healthy osteoblasts independent of ER stress. Therefore, autophagy stimulation could dampen osteoblast ISR by clearing misfolded procollagen to improve proteostasis, recycling cellular components to improve amino acid availability, and removing dysfunctional mitochondria to reduce reactive oxygen species and maintain energy homeostasis. In support of this, *Tfeb* elevation in osteoblasts reduced expression of stress responsive genes such as *Malat1* (Metastasis Associated Lung Adenocarcinoma Transcript 1).

An increase in osteoprogenitor proliferation and their differentiation into osteoblasts could also increase bone formation. Indeed, our ex vivo studies suggest that *Tfeb* elevation in the osteoblast lineage increases their proliferation. Moreover, *Tfeb* elevation increased expression of genes related to bone development, matrix production, and mineralization in a population of periosteal mesenchymal cells that we hypothesize to be osteoprogenitors. Together, these results suggest that *Tfeb* elevation may enhance bone formation by increasing the proliferation and differentiation of osteoprogenitors.

One limitation of our study is that we did not prove that the observed effects are exclusively due to increased autophagy. We used multiple approaches to show that *Tfeb* elevation enhances autophagy in osteoblasts. However, like all transcription factors, *Tfeb* can have functions in addition to stimulating autophagy in different cell types. To address this, we evaluated and ruled out two major alternative mechanisms by which *Tfeb* could influence bone formation. First, we ruled out possible stimulation of mitochondrial biogenesis by *Tfeb* by demonstrating that mitochondrial biogenesis genes (scRNA-seq results) or mitochondrial mass (ex vivo MitoTracker results) are not increased in response to elevated *Tfeb*. Second, we measured levels of sclerostin (an inhibitor of bone formation) in bones and serum and ruled out increased lysosomal degradation of sclerostin as a potential mechanism. Taken together, our evidence of increased autophagy, the exclusion of these alternative pathways, and the fact that *Tfeb* activation produces phenotypes opposite to those resulting from autophagy loss, we concluded that the observed skeletal effects are most consistent with autophagy stimulation. Nonetheless, further studies in which *Tfeb* is elevated in macroautophagy-deficient osteoblast lineage are needed to rule out the contributions from other lysosomal functions or degradative pathways (e.g., microautophagy, chaperone-mediated autophagy, endocytosis) to the anabolic impact of *Tfeb* elevation.

In summary, elevating *Tfeb* in osteoblast-lineage cells stimulates autophagy and promotes bone formation, leading to increased bone mass and strength. Several important questions remain, including: (i) which of the cellular changes we observed are responsible for the observed anabolic effects, (ii) what molecular mechanisms underlie these effects, and (iii) whether stimulating *Tfeb* or autophagy would be beneficial in skeletal disorders characterized by dysfunctional bone formation. Addressing these questions is critical for evaluating the therapeutic potential of *Tfeb* or autophagy activation for skeletal diseases.

## Abbreviations

ER: endoplasmic reticulum
ISR: integrated stress response
rRNAs: ribosomal RNAs
scRNA-seq: single-cell RNA sequencing
*Tfeb*/TFEB: transcription factor EB
